# Proximity Profiling of the CFTR Interaction Landscape in Response to Orkambi

**DOI:** 10.3390/ijms23052442

**Published:** 2022-02-23

**Authors:** Melissa Iazzi, Audrey Astori, Jonathan St-Germain, Brian Raught, Gagan D. Gupta

**Affiliations:** 1 Department of Chemistry and Biology, Ryerson University, Toronto, ON M5B 2K3, Canada; melissa.iazzi@ryerson.ca; 2 Department of Medical Biophysics, University of Toronto, Toronto, ON M5S 1A1, Canada; astori.audrey@gmail.com (A.A.); jonstgermain@gmail.com (J.S.-G.); brian.raught@uhnresearch.ca (B.R.)

**Keywords:** CFTR interactions, CFTR modulators, cystic fibrosis, Orkambi, theratyping, protein trafficking, chaperones

## Abstract

Deletion of phenylalanine 508 (∆F508) of the Cystic Fibrosis Transmembrane Conductance Regulator (CFTR) anion channel protein is the leading cause of Cystic Fibrosis (CF). Here, we report the analysis of CFTR and ∆F508-CFTR interactomes using BioID (proximity-dependent biotin identification), a technique that can also detect transient associations. We identified 474 high-confidence CFTR proximity-interactors, 57 of which have been previously validated, with the remainder representing novel interaction space. The ∆F508 interactome, comprising 626 proximity-interactors was markedly different from its wild type counterpart, with numerous alterations in protein associations categorized in membrane trafficking and cellular stress functions. Furthermore, analysis of the ∆F508 interactome in cells treated with Orkambi identified several interactions that were altered as a result of this drug therapy. We examined two candidate CFTR proximity interactors, VAPB and NOS1AP, in functional assays designed to assess surface delivery and overall chloride efflux. VAPB depletion impacted both CFTR surface delivery and chloride efflux, whereas NOS1AP depletion only affected the latter. The wild type and ∆F508-CFTR interactomes represent rich datasets that could be further mined to reveal additional candidates for the functional rescue of ∆F508-CFTR.

## 1. Introduction

Cystic fibrosis (CF) is a fatal inherited disease caused by mutations in CFTR that lead to varying clinical manifestations and severity [1,2]. The Cystic Fibrosis Transmembrane Conductance Regulator (CFTR) protein resides at the apical membrane of epithelial cells and functions as an ion channel that mediates the flux of chloride and bicarbonate ions [3,4]. Despite the development of therapeutic regimens, patient quality of life remains limited and even the most successful compounds do not achieve wild type (WT) level conductance [5,6]. The ∆F508 deletion mutation accounts for the majority of the CF population, making up approximately 70% of all patients, although its prevalence varies depending on the geographical origin of the patients [7,8]. ∆F508-CFTR is a class II mutation known for its trafficking defect caused by misfolding and ER retention and degradation [8]. In 2015, ivacaftor-lumacaftor (Orkambi) was approved for use in patients aged 12 years or higher and homozygous for the ∆F508 mutation and was recently extended to include those aged 6–11 years old [7,9]. Orkambi is a combination treatment consisting of a small molecule corrector compound (VX-809, lumacaftor) that promotes protein stability and forward trafficking [10], and a small molecule potentiator (VX-770, ivacaftor) that promotes the open state of the channel [10]. Ivacaftor (Kalydeco) is also approved for use on its own for the treatment of for CF patients aged four months or older with a gating variant [7]. Orkambi is associated with a variable clinical response [11,12].

The CFTR protein interaction network, and how it may be modulated by treatment, is poorly understood. This knowledge may help to guide theratyping efforts and provide co-targeting strategies for improving precision drugs that target CFTR alone [13]. To this end, proximity-dependent biotin identification (BioID) was used to characterize the interactomes of the WT and ∆F508-CFTR. BioID allows for the capture of ‘neighbourhood’ proteins in the context of a living cell and provides data that are complementary to more established techniques such as affinity purification mass spectrometry (AP-MS) but with the added benefit of enriching for low affinity interactions and avoiding post-lysis artefacts [14]. The N-terminus of each construct was fused with a mutant form of an *E. coli* biotin conjugating enzyme, BirA R118G (BirA*) [14]. BirA* activates free biotin to the highly reactive intermediate biotinyl-5′-AMP, which covalently reacts with lysine residues in proximal proteins within a ~10 nm radius surrounding each protein of interest, or “bait” [15]. Most human cells do not express de-biotinylase, therefore the biotinylation of proximal proteins is a permanent reaction, and the labelling over 16 h (approximately the length of a cell cycle), allows for the amplification of signal from these cycling interactions [14]. Since the biotin ‘tag’ is covalently attached, the cells can endure lysis under harsh buffer conditions to efficiently solubilize all membranes and organelles. These proximal proteins, or “prey”, can then be captured and isolated using streptavidin linked to sepharose beads and identified by mass spectrometry [14]. It has been suggested that over half of the variation observed in lung function could be due to non-CFTR modifier genes, highlighting the need to better understand the WT CFTR interactome and how it changes in response to drug treatment [16]. Using BioID, we identified known and unknown associations in the WT CFTR and ∆F508-CFTR interactomes, as well as those that are modulated in ∆F508-CFTR upon exposure to Orkambi treatment.

## 2. Results

### 2.1. BioID Identifies a Proximity Interactome for WT CFTR

FLAG-BirA*-CFTR WT and mutant fusion constructs were expressed in HEK293 Flp-In T-REx cells [17]. Each CFTR open reading frame was cloned in-frame with an N-terminal FLAG-BirA*. The system enables tetracycline-inducible expression of the transgenes expressed at a single copy (from the same Flp Recombination Target containing locus) through a Flp-mediated recombination event. Endogenous CFTR levels in this cell line are likely to be very low, as indicated by RNAseq data [18].

**Figure 1:**
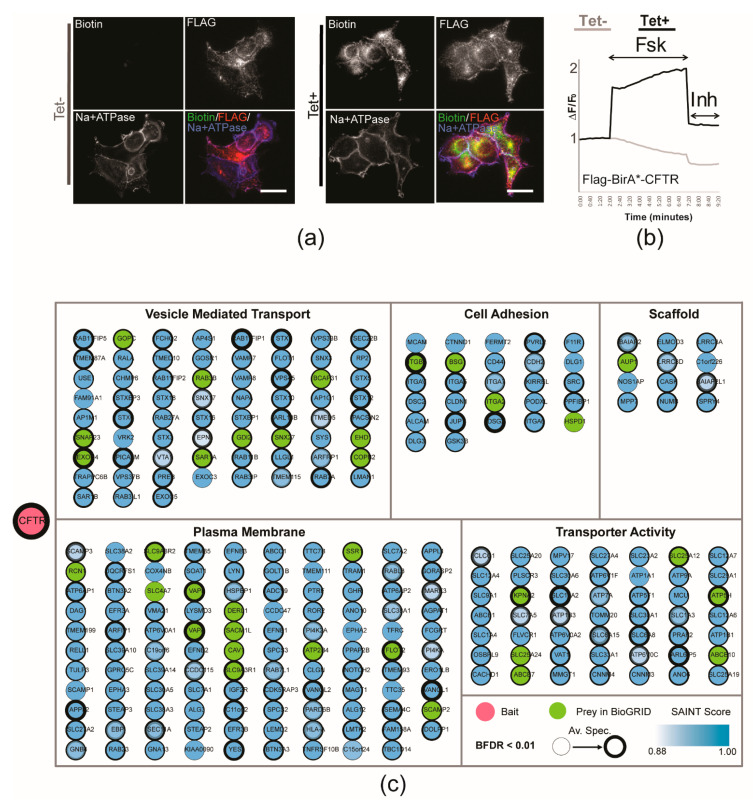
GO enrichment analysis of plasma membrane localized FLAG-BirA*-CFTR. (**a**) Immunofluorescence images of FLAG-BirA*-CFTR supplemented with tetracycline and biotin, for 24 h. Staining was performed with fluorophore-conjugated streptavidin (green), anti-FLAG (red), and Na+/K+ ATPase (blue) as a plasma membrane marker. FLAG-BirA*-CFTR is seen to be localizing at the plasma membrane and high levels of biotinylation are only detected in conditions supplemented with biotin. (**b**) The cells were treated with the tetracycline for 24 h before FLIPR functional assay. CFTR was stimulated using Fsk. CFTR-mediated depolarisation of the plasma membrane was detected as an increase in fluorescence following which CFTRinh-172 was added to inactivate CFTR. (**c**) Network representation of BioID data from HEK293 T-REx cells, with “known interactor” (BioGRID) highlighted in green. There are a total of 474 high confidence ‘preys’ with a BFDR ≤ 0.01. Preys categorized using Gene Ontology (GO) enrichment for key cellular components or functions. List of genes in GO annotation map can be found in Supplementary Dataset S1.

Upon tetracycline induction (Supplementary Figure S1a), the FLAG-BirA*-CFTR fusion protein (~200 kDa) colocalized with the plasma membrane (PM) marker Na/K ATPase (Figure 1a). CFTR-dependent chloride efflux was assessed in these cells using the fluorometric imaging plate reader membrane depolarization (FLIPR) assay. This assay has been extensively used to assess functional responses of CFTR by detection of rapid changes in membrane potential [19]. The spike in fluorescence observed after the addition of the cAMP agonist forskolin (Fsk) in cells expressing FLAG-BirA*-CFTR (Figure 1b) is similar to what has been observed with WT CFTR [20]. The increase is related to FLAG-BirA*-CFTR activity as it is completely inhibited upon the addition of a specific CFTR inhibitor (inh-172) [21]. Furthermore, uninduced cells that do not express the fusion construct do not respond to forskolin and inh-72, respectively (Figure 1b). The BirA* is attached to the N-terminus of CFTR with a flexible linker to mitigate interference of the BirA* on CFTR processing and interactions. The FLAG-BirA*-CFTR fusion is able to retain its ability to localize to the PM and perform chloride efflux, and was therefore selected for BioID implementation. Similarly sized N-terminal GFP and YFP-fusions of CFTR have been utilized to report on CFTR trafficking and function previously [21,22].

The proximity interactome for WT CFTR comprised 474 high confidence proximity interactors (BFDR ≤ 0.01). Statistically enriched Gene Ontology (GO) categories included Vesicle Mediated Transport (67; GO:0016192), Cell Adhesion (28; GO:0007155), Scaffold (13; PC00226), Plasma Membrane (109; GO:0005886), and Transporter Activity (49; GO:0005215) [23]. Fifty-seven previously validated CFTR-interactors were identified (BioGRID database; Figure 1c and Supplementary Dataset S1) and 22 additional proximity interactors overlapped with manually curated meta-analyses that collated 179 CFTR predicted interactors from several high-throughput screens [24,25,26]. Additionally, 33, 27, 17 and 38 of our high-confidence proximity interactors were also seen in AP-MS studies performed in bronchial epithelial [8,27,28] or HEK293 [29] cells, respectively (Supplementary Figure S1b and Supplementary Dataset S2).

Previously validated CFTR interactors in our dataset included: both isoforms of a Na+/H+ exchanger regulatory factor (NHERF or SLC9A3R1/2) which anchors CFTR to the actin cytoskeleton through a multiprotein complex [30,31]; Golgi Associated PDZ Additionally, Coiled-Coil Motif Containing (GOPC) also known as the CFTR-associated ligand (CAL) [32]; and the integral membrane protein Lemur Tyrosine Kinase 2 (LMTK2), which affects CFTR activation [33]; Ubiquitin Specific Peptidase 19 (USP19), which rescues ∆F508del-CFTR when overexpressed [34] and Golgi Reassembly Stacking Protein 2 (GORASP2), which mediates unconventional CFTR trafficking [35] (Supplementary Dataset S1). Notably, all 10 subunits of the Endoplasmic Reticulum Membrane Protein Complex (EMC) were identified as high confidence proximity interactors with FLAG-BirA*-CFTR. The EMC is an insertase that chaperones the co-translational membrane insertion and folding of multipass membrane proteins (Supplementary Figures S1c and S3c) [36].

Since BioID provides a history of proximal associations of the tagged protein over approximately the length of a cell cycle, we could manually curate the known or predicted localizations of several identified preys to demarcate the trafficking route of CFTR as it is processed and enters into vesicular pathways to, and from, the PM (Supplementary Figure S1c). Pairwise comparison with a recent large scale BioID dataset comprising 192 representative baits from all major cellular compartments [37] revealed that FLAG-BirA*-CFTR prey profiles most closely resemble that of a PM-localized, membrane trafficking bait protein (Supplementary Figure S2a).

### 2.2. ∆F508-CFTR Interactome and Profiling of Orkambi Response

**Figure 2:**
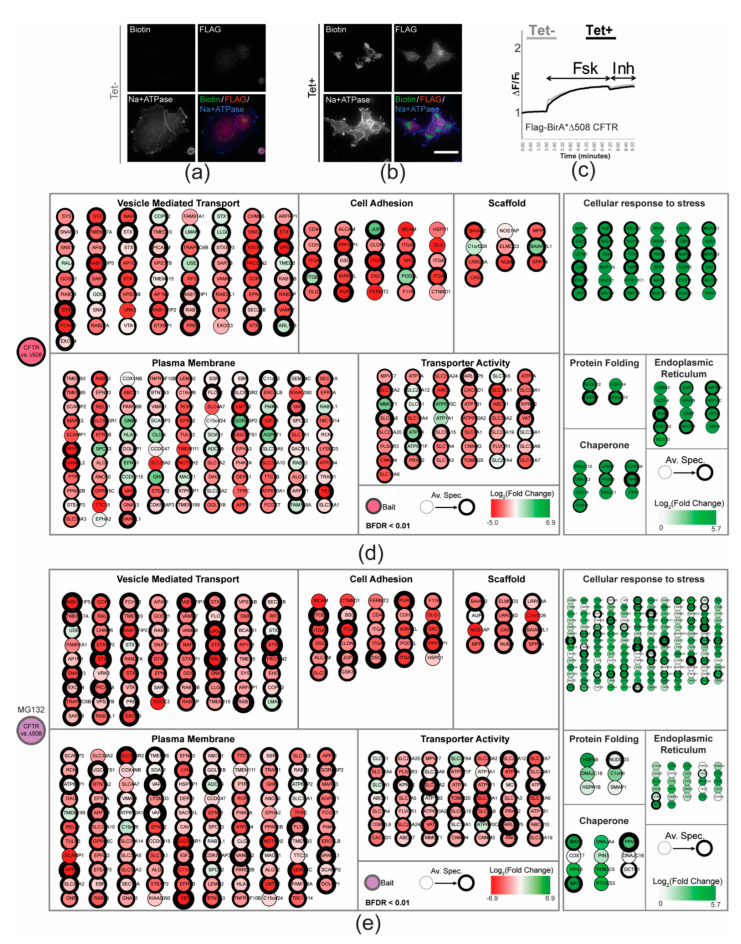
GO enrichment analysis of intracellularly localized FLAG-BirA*-∆F508-CFTR. (**a**,**b**) Immunofluorescence images of FLAG-BirA*-∆F508-CFTR without (**a**) or with (**b**) 1 µg/mL tetracycline, and supplemented with 50 µM biotin for 24 h. Labelling was performed with fluorophore-conjugated streptavidin (green), anti-FLAG (red), and Na+/K+ ATPase (blue) as a plasma membrane marker. (**c**) The cells were treated with the tetracycline for 24 h before FLIPR functional assay (see Materials and Methods). CFTR was stimulated using Fsk, and the CFTR-dependent activity was determined by sensitivity to CFTRinh-172 (Inh). The peak changes in fluorescence to CFTR agonists were normalized relative to the baseline fluorescence (ΔF/F0). (**d**) WT CFTR interactome filtered ‘preys’ with a BFDR ≤ 0.01. Preys categorized using Gene Ontology (GO) enrichment for key cellular components or functions. The thickness of the border represents an increasing average peptide count. The node colour reflects the log 2-fold change (log_2_FC) from WT to the normalized ∆F508 mutant condition. The darker red nodes represent greater negative fold change. The darker green nodes represent greater positive fold change. (**e**) Using the same colour scheme as in (**d**), categorized preys comparing WT to ∆F508-CFTR + MG132 condition were coded according to log_2_FC and enriched GO terms.

Upon tetracycline induction, FLAG-BirA*-∆F508-CFTR protein did not traffic to the PM (Figure 2a,b), and chloride efflux activity was low in FLAG-BirA*-∆F508-CFTR expressing cells (Figure 2c). The increase in fluorescence observed after the addition of forskolin (~50% less than FLAG-BirA*-CFTR cells) was unaffected by the addition of inh-172, and was also observed in the uninduced line, suggesting that this was not due to CFTR activity (Figure 2c).

At the ER, CFTR is subject to extensive quality control mechanisms—due to the ∆F508-CFTR folding defect, it results in premature degradation of up to 99% of the mutant protein [8,38]. Treatment with proteasome inhibitor carbobenzoxy-Leu-Leu-leucinal (MG132) [39] presumably stabilized the mutant bait protein, as we detected ~2× higher levels of self-labelled BirA*-∆F508-CFTR peptides in BioID experiments, as assessed by mass spectrometry (Supplementary Figure S3b). BioID was performed on FLAG-BirA*-∆F508-CFTR expressing cells in the absence or presence of MG132, and these interactomes constituted 63 and 260 high confidence interactors, respectively (Figure 2d,e and Figure S4). For comparison to the WT CFTR BioID, we normalized the ∆F508-CFTR dataset using average bait spectral counts (Supplementary Figure S3b), and this normalized ∆F508-CFTR interactome constituted 626 high confidence interactors (Supplementary Table S7). GO enrichment analysis indicated a significant reduction in interactions with proteins categorized as PM-localized or with assigned functions at the cell surface (Figure 2d,e). By contrast, a gain of interactors for untreated and MG132 treated interactomes was observed in the GO categories: Cellular Response to Stress (238 and 165; GO:0033554), Protein Folding (45 and 6; GO:0006457), Endoplasmic Reticulum (28 and 27; GO:0005783), and Chaperone (31 and 11; PC00072) (Figure 2d,e; Supplemental Dataset S6) [23]. These category constituent changes were not impacted by normalization, as similar trends were seen in the non-normalized ∆F508-CFTR interactome (Supplementary Figure S3a). Correspondingly, pairwise comparison with 192 BioID baits representing all major cellular compartments reveals that the ∆F508-CFTR bait profile overlaps most with membrane chaperone proteins in the ER tagged at their cytosolic, but not lumenal domains (Supplementary Figure S2b). Additionally, 165 preys categorized under ‘Cellular Response to Stress’ formed the bulk of the gain of interactors due to MG132 addition and included several components of the proteasome machinery (Figure 2e and Supplementary Dataset S4). Several previously validated CFTR interactions were lost or decreased upon mutation in our dataset. We detected loss of the CFTR- NHERF-1 interaction in the ∆F508 interactome, which has also been previously reported [31]. CFTR, when normally trafficked, accumulates in clathrin coated vesicles and in early endosomes [30]. Strikingly, 21 preys associated with clathrin coat machinery (GO:0030136) in the CFTR interactome were not detected in the ∆F508-CFTR counterpart (Supplementary Dataset S3).

**Figure 3:**
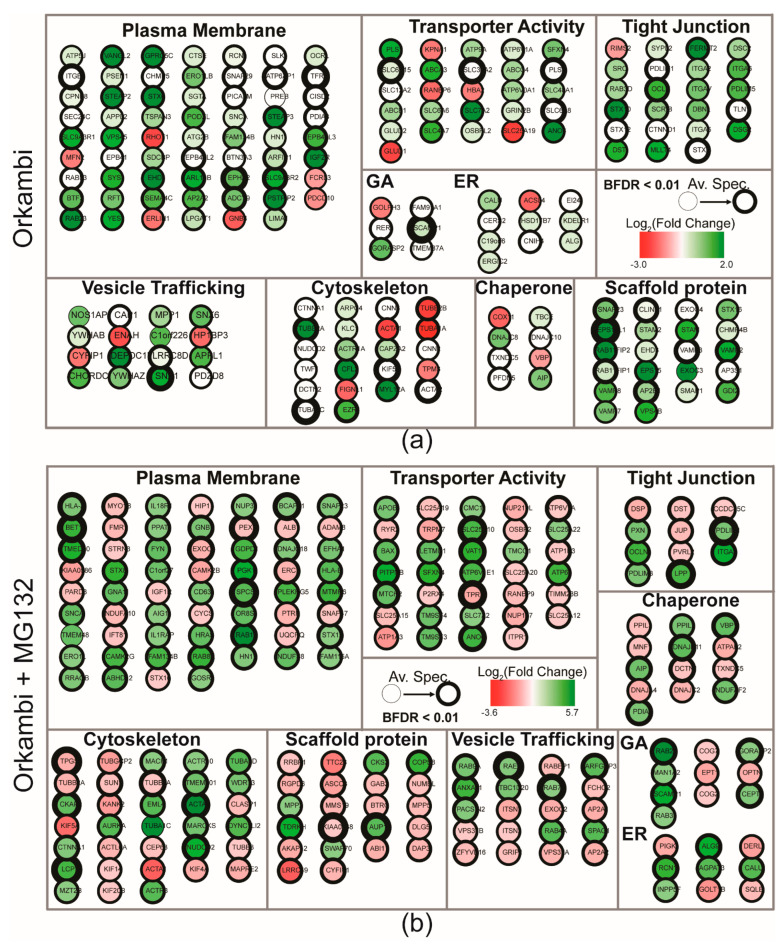
GO enrichment analysis of FLAG-BirA*-∆F508-CFTR after exposure to Orkambi. (**a**) Manually curated GO-enrichment analysis of proximity interactors considered significantly gained or lost upon exposure to Orkambi combination therapy (3 µM VX-809 + 1 µM VX-770). The thickness of the border represents an increasing average peptide count. The darker green nodes represent a larger increase in normalized spectral counts associated with the prey. List of genes in GO annotation map can be found in Supplementary Dataset S1. (**b**) GO-enrichment analysis of proximity interactors considered significantly gained or lost upon exposure to MG132 and Orkambi combination therapy (3 µM VX-809 + 1 µM VX-770). List of genes in GO annotation map can be found in Supplementary Dataset S1.

BioID interactomes for ∆F508-CFTR in Orkambi-treated cells (in the absence or presence of MG132) constituted 127 (531 normalized) and 290 high confidence proximity interactors, respectively (Supplementary Dataset S7). Notably, we detected 45 (253 normalized) and 43 ‘restored’ interactions (untreated or MG132 treated, respectively), with Orkambi treatment of ∆F508-CFTR, which were present in the WT CFTR interactome, and enriched in GO categories associated with PM and function in cell surface activities (Figure 3a,b, Supplementary Dataset S6, S3a and S5). Additional ‘restored’ interactors included: Calumenin (CALU), a CFTR chaperone with decreased expression in cells expressing ∆F508-CFTR, but which can be reversed by rescuing-CFTR trafficking to the PM [40,41]; Synaptosome Associated Protein 23 (SNAP23), which binds to CFTR and inhibits its activity in the presence of Syntaxin 1A (STX1A) and is thought to regulate CFTR gating at the PM [42]; a soluble N-ethylmaleimide-sensitive factor attachment receptor (SNARE) protein, syntaxin 6 (STX6), which localizes to the trans-Golgi network where it interacts with CFTR via the PDZ domain-containing protein, GOPC, to form a functional complex [43]; and STIP1 Homology and U-Box Containing Protein 1 (STUB1) is among several proteins that facilitate the ubiquitination of misfolded CFTR [44].

### 2.3. Comparison of CFTR and ∆F508-CFTR Interactomes Reveal Orkambi Responsive and Non-Responsive Interactions

**Figure 4:**
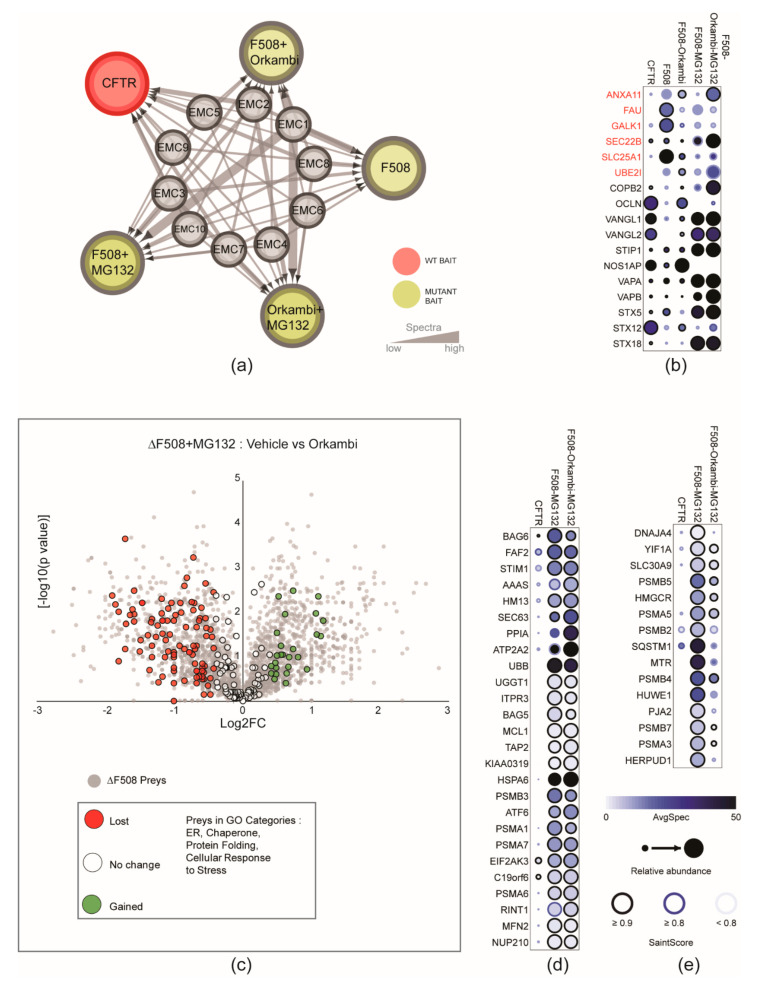
Trends within the proximity interactomes for WT and ∆F508-CFTR. (**a**) BioID network of ER Membrane Protein Complex (EMC). All 10 EMC subunits were identified in each of the 5 WT and ∆F508-CFTR datasets. Bait and edges are colour-coded as indicated in the legend. Edge thickness is proportional to total peptide counts. (**b**) Normalized spectral abundance dot plot displaying selected prey profiles across different baits along with corresponding SAINT scores. Displayed is a snapshot of preys that have changes in abundance from ∆F508 and/or Orkambi exposed conditions, and with or without MG132. Preys highlighted in red are known modifiers of ∆F508 CFTR [46]. (**c**) Volcano plot of significance versus log_2_FC on the y and x axes, respectively, comparing biotinylated proteins identified in ∆F508 + MG132 vehicle to Orkambi exposed conditions. Preys marked in red have higher spectral counts in the ∆F508 + MG132 condition from Figure 2e illustrating preys enriched in GO categories for ER, chaperone, protein folding, and cellular response to stress. (**d**,**e**) Displayed dot plot is a subset of the enriched mutant preys in GO categories for ER, chaperone, protein folding, and cellular response to stress, that show no or marginal (**d**), or significant (**e**) response to Orkambi treatment.

A notable finding was that all 10 EMC subunits were detected as high confidence proximity interactors in both the WT and mutant interactomes (Figure 4a and Figures S1c and S3c). The EMC complex has been implicated in the biogenesis of CFTR [45], but has not been detected in previous interactomic studies. We next focused on a subset of preys for which spectral counts were significantly changed after treatment with Orkambi in the ∆F508-CFTR dataset and suggest these to be candidates for further study and highlight several here (Figure 4b). The changes we observed were likely not due to differences in expression of the bait proteins, as the trends are present in raw or normalized datasets (Supplementary Figure S3a,d). Notably, the knockdown of several proteins (FAU, ANXA11, GALK1, SEC22B, SLC25A1, UBE2EI) in this subset has been shown to rescue ∆F508-CFTR functional activity, with little effect on WT CFTR [46]. Consistent with this, our data indicates that all six of these prey spectral counts are specifically elevated in the ∆F508 interactome, and attenuated by Orkambi addition (Figure 4b). Depletion of COPB2 (COPI Coat Complex Subunit Beta 2), also known as beta-COP, has been shown to impair CFTR trafficking to the PM and it is speculated that ∆F508-CFTR is a COPI cargo for retrograde transport to the ER [47]. OCLN (Occludin) is a tight junction protein, and its transcript levels are reduced in CFTR knockout mice [48]. Additionally, associations with the planar cell polarity components VANGL1/2 have implications for the apical polarity of CFTR, airway development, and disease [49]. Stress-induced phosphoprotein 1 (Stip1) is among several proteins that modulate ∆F508-CFTR folding and PM density [50]. VAMP-Associated Proteins (VAPA/B) have been suggested to regulate CFTR biogenesis in the ER [51]. Syntaxin 5 (STX5) overexpression has been shown to stimulate unconventional trafficking of core-glycosylated ∆F508-CFTR to the PM [35]. Syntaxin-17 (STX17), a SNARE protein, interacts with CFTR [52] and the loss of the CFTR-STX17 interaction impairs bacterial clearance and could play a critical role in infectious diseases among CF patients. Syntaxin-12 (STX12) and STX6 form a SNARE complex that regulates transport between late endosomes and the trans-Golgi network. These preys have also been identified as interactors of STX17 [53]. Overexpression of Syntaxin 18 (STX18) has been shown to generate more ER exit sites (ERES) [54]. ERES facilitate the formation and function of COPII complexes [55]. Response to STX18 overexpression could potentially increase the abundance of COPII complexes, which may in turn promote ∆F508-CFTR exit from the ER [55,56].

To select for candidates that could be involved in the processing, stabilizing or folding functions of the ∆F508-CFTR mutant baits, we focused on preys that belonged to the GO categories that we defined earlier (Cellular Response to Stress, Protein Folding, Endoplasmic Reticulum, and Chaperone; Figure 2e). These were mapped on a volcano plot according to their spectral fold change (or lack thereof) upon Orkambi treatment in the presence of MG132 (Figure 4c), and several are highlighted here in dot plots (Figure 4d,e). Considering the subset of ∆F508-CFTR preys which do not appear to change in spectral counts upon Orkambi treatment concomitant with proteasome inhibition (Figure 4d), these may represent interactions that may be co-targeted to improve the efficacy of this drug. For example, BAG5 and BAG6 proteins appear to selectively associate with the ∆F508 mutant, and these associations persist with Orkambi (Figure 4d). To date, six human Bcl2-associated athanogene (BAG 1–6) proteins have been identified and previous work has shown that the knockdown of BAG1 and BAG3 leads to the functional correction of ∆F508-CFTR [57]. BAG2 stimulates the chaperone-assisted maturation of CFTR by inhibiting the ubiquitin ligase activity of CHIP [58]. Only BAG 1–3 have been characterized with respect to the nature of their interaction with CFTR in both its WT and mutant state. BAG5 and BAG6 similarly have been shown to exhibit Hsp70-inhibitory activity, however, their relationship with CFTR has not been studied [57]. A second example from this group is Stromal Interaction Molecule 1 (STIM1; Figure 4d). Elevated intracellular Ca^2+^ levels can lead to the lack of functional CFTR in airway epithelial cells [59]. Store-operated Ca^2+^ entry is an essential mechanism for regulating Ca^2+^ homeostasis driven by the interaction between STIM1 and Calcium Release-Activated Calcium Modulator 1 (ORAI1) [60]. CFTR forms a molecular complex with transient receptor potential canonical 6 (TRPC6) which is lost in CF leading to an influx of TRPC-6 dependent Ca^2+^ through ORAI1 [61]. The Orkambi non-responsive association we observe between STIM1 and ∆F508-CFTR could be of physiological relevance, since decreased Ca^2+^ levels have also been associated with the correction of ∆F508-CFTR [62]. A third example is Sarco/Endoplasmic reticulum Ca^2+^ ATPase (SERCA) 2; also known as ATP2A2 (Figure 4d). CALU modulates the expression of SERCA pump activity in non-CF and CF bronchial epithelial cells [42]. Enhanced SERCA pump activity has been shown to increase ER retained Ca^2+^ and has been correlated with a decreased interaction between SERCA2 and ∆F508-CFTR compared to WT CFTR [42]. This is another association that is non-responsive to Orkambi and may be a candidate for further examination to enhance functional rescue of ∆F508-CFTR.

We also note the subset of Orkambi-restored interactions of ∆F508-CFTR that would be candidates for further study (Figure 4e and Figure S4). Solute Carrier protein family 30A9 (SLC30A9) is a nuclear receptor coactivator involved in the transcriptional regulation of Wnt-responsive genes [63]. Wnt signalling has been shown to be impaired in CFTR mutants but can be restored when WT CFTR is overexpressed [64]. We also detect higher spectral counts of the sequestosome 1 (SQSTM1) protein with ∆F508-CFTR, which are reduced upon Orkambi treatment (Figure 4e). Recent work has shown that defective ∆F508-CFTR leads to small ubiquitin like-modifier (SUMO)ylation activation of tissue transglutaminase (TG2), resulting in proteasome degradation and accumulation of SQSTM1 [65]. Correspondingly, the depletion of SQSTM1 can favour the trafficking of ∆F508-CFTR protein to the epithelial cell surface [66], which parallels our findings here.

Our data also show that PSMA3, PSMA5, PSMB2, PSMB4, and PSMB5 (all subunits of the 20S core proteasome complex) are found preferentially associated with ∆F508-CFTR upon MG132 treatment (Figure 4e), and these interactions are attenuated upon Orkambi treatment (Figure 4e). This observation is consistent with an earlier finding that chaperone association with ∆F508-CFTR is attenuated by VX-809 [67]. It has been reported that Hsp40 co-chaperones, referred to as J proteins, interact with CFTR during its initial translation stages [68]. DNAJ proteins may serve as pro-degradation components of the quality control machinery and of ∆F508 specifically [68] (Supplementary Figure S3d). We therefore surveyed all co-chaperone DnaJ (Hsp40) and Hsp70/90 chaperone members in our dataset (Supplementary Figure S5a). Previously characterized DnaJ members DJA1 and DJA2 were identified in our datasets (Supplementary Dataset S5) and have been previously reported to promote folding of CFTR but display contradictory functions which could be due to their differences in binding to CFTR [69]. In addition to these, we detect two other members of this family, DNAJA3 and DNAJA4 (Figure 4e and Figure S5a). Overall, we note a striking increase in chaperone associations with the ∆F508-CFTR bait, as compared to the WT, which is consistent with the stabilizing function of this protein family [68]. Additionally, Orkambi attenuates ∆F508 mutant associations in 18 of 24 chaperones (Supplementary Figure S5a), which is consistent with previous corrector data [67]. Interestingly the presence of MG132 abrogates the normal effect of Orkambi on Hsp chaperone associations in many Hsp 70/90 family members, but unequally in Hsp 40 members (Supplementary Figure S5a).

### 2.4. Proximity Interactions That Affect Trafficking and/or Function of CFTR

**Figure 5:**
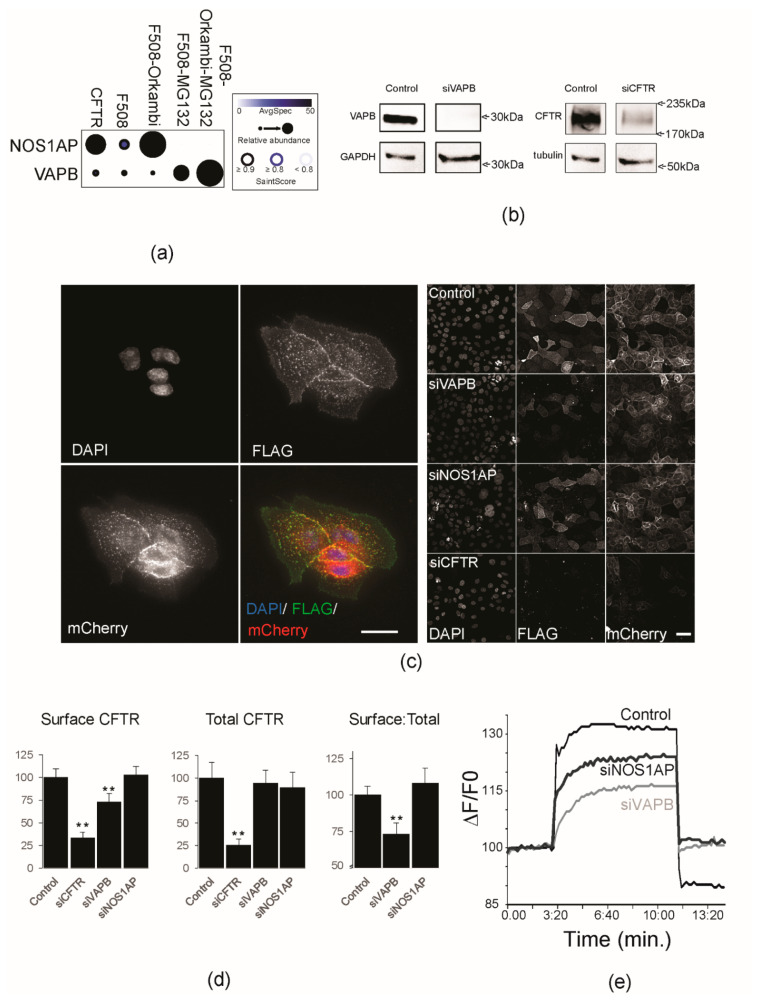
Proximity interactions affect PM density and function of CFTR. (**a**) Dot plot of normalized prey spectral counts and relative abundance across baits for NOS1AP and VAPB. (**b**) Western blots showing levels of WT CFTR and VAPB in control or siRNA treated cells, with GAPDH or α-tubulin as a loading control. Reference molecular weight markers are indicated on the right of each blot. (**c**) Left panel: Representative grayscale micrographs of CFBE mCherry-Flag-WT-CFTR cells illustrate nuclear (DAPI), surface CFTR (FLAG) and total CFTR (mCherry) fluorescence levels, with a psuedocoloured merge image. Bar = 10 µm. Right panel: Representative grayscale micrographs of single fields of cells for each channel after 96 h siRNA knockdown as indicated (Control; siVAPB, siNOS1AP and siCFTR, respectively). Micrographs are scaled equally for each channel. Bar = 20 µm. (**d**) Quantification of Surface CFTR (left panel), Total CFTR (middle panel) levels, and Surface:Total ratios (right panel) for each knockdown. Values are normalized to the control and shown as the mean of three experiments with >300 cells counted for each experiment. Error bars denote standard error, ** denotes *p* < 0.01 with Student’s *t*-test. (**e**) Representative traces of control, siNOS1AP, or siVAPB-treated cells assayed for CFTR-dependent chloride efflux using FLIPR (see Materials and Methods). Fsk was added to stimulate CFTR and deactivated with CFTRinh-172.

A key criterion for successful CF therapy is the restoration of an adequate steady state concentration of functional CFTR on the surface of airway cells. It is therefore important to characterize candidate interactors with respect to CFTR levels at the PM and on CFTR channel conductance activity. Our BioID data show the presence of VAMP-Associated Proteins (VAPA/B) and Nitric Oxide Synthase 1 Adaptor Protein (NOS1AP) in all WT and ∆F508-CFTR datasets (Figure 4b and Figure 5a). VAPs have been proposed to regulate CFTR biogenesis in the ER [49]. NOS1AP is a direct interactor of NOS1, which has been associated with CF disease phenotypes [70,71]. Notably NOS1AP association with WT CFTR was altered in the mutant bait, and restored in the presence of Orkambi (Figure 5a). In addition, no association with ∆F508-CFTR was detectable in the presence of MG132. Conversely, VAPB association with ∆F508-CFTR was enhanced by MG132, and Orkambi had a marginal effect regardless of MG132 status (Figure 5a).

To determine the importance of CFTR’s interaction with these candidates, we knocked down CFTR (siCFTR), NOS1AP (siNOS1AP), and VAPB (siVAPB) in the well characterized CFBE reporter cell line (Figure 5b) [72,73,74]. The reporter comprises a FLAG epitope tag on an extracellular loop region of CFTR and a fluorescent mCherry moiety located on the cytoplasmic side under an inducible promoter (Figure 5c) [73]. The double-tagged constructs allow for the simultaneous readout of the total protein expressed in the cell and the fraction at the PM. This allows us to estimate traffic efficiency based on ratiometric parameters that normalize for different expression levels of the reporter (see Materials and Methods). After expression induction of mCherry-Flag-WT-CFTR, cells were fixed and imaged to measure the total CFTR and CFTR at the PM (‘surface’) using a custom-written MATLAB script that determines pixel intensity values in the corresponding channels for each cell, as well as a surface:total ratio (see Materials and Methods; Figure 5c,d). As a control, treatment with siCFTR significantly decreased (~70–80%) the total and surface fluorescence CFTR reporter signal (Figure 5c). Treatment with siNOS1AP did not significantly alter the levels of total or surface mCherry-Flag-WT-CFTR, or the surface:total ratio, suggesting no major effect on trafficking (Figure 5d). By contrast, siVAPB knockdown significantly reduced (*p* < 0.01) surface CFTR levels by ~30% while not significantly altering total mCherry-Flag-WT-CFTR levels, as reflected in the lower normalized surface:total ratio (Figure 5d).

Inducible FLAG-BirA*-CFTR HEK293 cells were treated with siNOS1AP and siVAPB to assess their impact on CFTR conductance using the FLIPR assay (see Materials and Methods). This strategy also allows for quantifying the role of individual genes in affecting CFTR function by inducing CFTR expression only after the gene of interest has been knocked-down. Representative traces of WT CFTR-dependent chloride efflux were generated from control, siNOS1AP, and siVAPB conditions (Figure 5e). Both siNOS1AP and siVAPB-treated cells exhibited a significant decrease (~50% and ~80% of control, respectively) in forskolin-stimulated CFTR activity.

## 3. Discussion

The BioID results established a comprehensive proximity interactome for WT CFTR as well as ∆F508-CFTR in vehicle, MG132, and Orkambi exposed conditions. A significant number of our high-confidence proximity preys in the WT dataset were established CFTR interactors (e.g., NHERF1, NHERF2, GOPC/CAL, LMTK2, USP19, GORASP2) [24]. When compared with a recent large scale BioID dataset comprising representative baits from all major cellular compartments [37], the CFTR BioID profile largely resembles that of PM anchored proteins such as KRAS but also membrane trafficking proteins such as ARF6 and RAB35 (Supplementary Figure S2a). Measurements of the endocytic rate of surface CFTR show that over 50% is internalized over a ten-minute period [75], so a large fraction of the CFTR pool actively cycles in transport routes that culminate in its steady state localization both intracellularly and at the cell surface. The N-terminal tagged Flag-BirA*-CFTR fusion likely adopts the predicted topology of CFTR as a multipass membrane protein with both its N and C termini facing the cytosol. Therefore, it is able to access cytosolic pools of biotinyl-AMP to label vicinal trafficking proteins that participate in its biosynthetic route. Recent work has identified the EMC as playing a vital role in the biogenesis of multipass transmembrane proteins containing destabilizing features, thereby alleviating the choice between function and stability [46]. Our data shows that all 10 EMC subunits were detected as proximity interactors in both WT and mutant interactomes, suggesting a critical role for CFTR biosynthesis. In a yeast phenomic model of ∆F508-CFTR, mutant CFTR biogenesis is impaired upon knockdown of the EMC [76]. EMC protein subunits have not been detected by AP-MS in prior studies likely due to the differences in interaction methodology. A number of factors could influence detectability in AP-MS, including the efficiency of solubilization of membrane inserted proteins or the degree of stability of the interaction. Our BioID data thus compliments the known AP-MS CFTR interaction landscape to include such associations, which may not be preserved post cell-lysis.

The mutant Flag-BirA*-∆F508 CFTR proximity profile demonstrates a marked reduction in preys associated with the PM or those involved in transporter activity and vesicle-mediated transport. By contrast, there is a significant gain of the preys corresponding to cellular response to stress, protein folding, endoplasmic reticulum, and chaperone classes. Our analysis of the CFTR and ∆F508 interactomes is consistent with the model that the trafficking defect caused by the ∆F508 deletion of CFTR leads to the dysregulation of a network of protein interactions needed for CFTR folding, trafficking to the PM, and enhances premature degradation [8]. The ∆F508-CFTR prey profile resembles several endoplasmic reticulum (ER)-membrane localized, cytoplasmic facing baits (Supplementary Figure S2b). Strikingly, there is little prey overlap with a C-terminally BirA* tagged ER protein (LRRC59) where the BirA* moeity is lumenal, but large overlap with its N-terminal tagged counterpart, where the BirA* is tagged to a cytosolic domain. We hypothesize that a (small) portion of BirA*-∆F508 is inserted into the ER membrane where BirA* can access cytosolic biotinyl-AMP pools, while the remainder is rapidly degraded by the ER quality control machinery. Consistent with this notion, treatment with Orkambi reveals significant restoration in preys associated with PM trafficking and function and which also overlap with our WT CFTR dataset. A large fraction of the chaperone, stress, and folding cohort of interactions of ∆F508 is also attenuated upon Orkambi treatment, thus partially resembling the WT state (Supplementary Figure S5a). In addition, several restored preys were found to be previously characterized interactors of CFTR (e.g., CALU, SNAP23, STX1A, STX6, GOPC, CLTC, STUB1; Supplementary Figure S5b). In general, since BioID integrates the proximal associations of a bait protein over an entire cell cycle [14], the size of the interaction space revealed by BioID versus AP-MS approaches can be difficult to compare directly [17,37,77,78]. Nevertheless, consistent with data from AP-MS interactomes of CFTR [8,27,28,29], we see a larger proximity interaction network for ∆F508-CFTR compared to WT CFTR, with the majority of ∆F508 interactors associated with protein folding and proteostasis pathways. Likewise, we also detect loss of several of these interactors upon corrector treatment, which results in a net smaller interactor count [28,29]. Overall, our BioID data therefore supports the existing interactomics model that off-pathway ∆F508 interactions consist of destabilized folding and degradation machinery [8], while standard WT interactions are PM trafficking and recycling related. Altogether, these findings are validative of our experimental strategy.

Proximity interactors of ∆F508-CFTR may be candidates for co-targeting with corrector drugs to improve the efficacy of rescue. A number of candidates that could play a role in ∆F508-CFTR rescue were identified (Figure 4). Notably, individual proteostasis and chaperone family member associations of ∆F508-CFTR were largely attenuated by Orkambi treatment (Supplementary Figure S5a), consistent with previous interactomic data [28,29]. However, the results from MG132 treated ∆F508-CFTR interactomes indicate that corrector drug effect on these associations may be context dependent, according to the sub-type specificity of the chaperone and the proteasomal activity. This supports the idea [79] that co-inhibiting chaperone function in ∆F508 mutants may enhance corrector efficacy.

VAPB and NOS1AP were identified as candidate CFTR modulators, and through the combination of FLIPR and membrane trafficking assays, we were able to assess channel function and surface delivery defects, respectively. VAPB knockdown results in a significantly reduced surface delivery and channel efflux of CFTR, and while we cannot at present rule out a direct effect on channel activity, our simplest interpretation is that reduced concentration of CFTR at the cell surface is the primary defect here. VAPB is an ER and Golgi-localized membrane anchored protein that participates in vesicle trafficking and regulates tethering at ER-contact sites via FFAT motifs of a number of lipid and proteostasis pathway components [49]. VAPB has been previously shown to inhibit degradation of ∆F508-CFTR by sequestering cytosolic degradation machinery [49], and in our study, ∆F508-CFTR association with VAPB is enhanced upon proteasome inhibition by MG132. We propose that VAPB knockdown results in increased targeting of the peripheral pool of CFTR to the proteasome, resulting in reduced surface levels and lower channel efflux. By contrast, NOS1AP knockdown decreases channel activity without affecting the surface levels of CFTR. Based on its reported function, NOS1AP and the associated nitric oxide (NO) Synthase 1 may act as CF modifiers, and possibly activate intracellular cAMP to impinge on CFTR channel function [70,80,81,82]. The NOS1AP association with ∆F508-CFTR is augmented by Orkambi treatment, and undetectable in MG132 treated cells. Thus, it is less likely to occur with proteasome associated pools of ∆F508 CFTR destined for degradation. We propose that NOS1AP associates with corrector accessible CFTR, possibly at the PM, where it may regulate channel activity. Further studies will be aimed at characterizing these associations in more detail and categorizing other candidates in these functional assays.

Despite the many benefits of using BioID to identify novel interactors, some limitations exist in this study. Firstly, due to the nature of ∆F508-CFTR retention in the ER and its rapid degradation by the quality control machinery, there may be less bait protein available (Supplementary Figure S3b). This may lead to a higher false negative rate of detection of preys in the ∆F508-CFTR dataset. This was partially mitigated by the use of MG132 in this study, and provided an additional comparison set from which to discriminate altered associations reliably. Secondly, our proximity profiles were generated in engineered HEK293 cells, an established cell model that has been extensively used for BioID analyses [17,37,77,78]. This cell line expresses very low levels of endogenous CFTR and is therefore routinely used to express heterologously expressed CFTR constructs. Unlike bronchial epithelial lines which can be suitable in vitro models for the human airway and for CF studies [73], these lines do not form apically differentiated epithelia and may not express several transcripts relevant to CF. However, CFTR interactome data generated in HEK293 cells (also in [29]) can be investigated further in more tissue-specific models, such as the CFBE lines we have employed here. That we have identified numerous previously validated CFTR interactors/regulators is proof of the utility of this approach. Future work would entail the use of orthogonal methods and CF cell lines to functionally validate and expand our dataset. The interactomes that we have generated can serve as a starting point for hypothesis driven studies for examining the modulation and dynamics of CFTR interactions. In conclusion, our work supports the continued use of BioID to study CFTR biology and suggests that it may also be effective in identifying important interactions in a variety of combinations of CF mutations and CFTR modulator drug contexts.

## 4. Materials and Methods

### 4.1. Cell Culture and Reagents

Cells were maintained in modified Eagle’s medium (DMEM) supplemented with 10% FBS, 100 g/mL penicillin/streptomycin at 5% CO_2_ at 37 °C. HEK293 T-REx cells were stably transfected with tetracycline-inducible pcDNA5 FRT/TO BirA-R118G—FLAG (BirA*FLAG) expression vectors, expressing ∆F508-CFTR.

### 4.2. Immunofluorescence and Immunoblotting

The following primary antibodies were used for IF experiments: mouse anti-FLAGm2 (Sigma Aldrich, Oakville, ON, Canada; at 1:500) and rabbit Na/K ATPase (Abcam, Cambridge, UK; at 1:1000). Streptavidin-488 (Abcam, Cambridge, UK; at 1:500) was used to detect biotinylated proteins in IF experiments and Streptavidin-HRP (Bio-Rad, Hercules, CA, USA; 1:5000) was used to detect biotinylated protein in immunoblotting experiments; both without a secondary antibody. Secondary antibodies used for IF were all obtained from Invitrogen, used at 1:1000, and include: Alexa Fluor 488 donkey anti-mouse and Alexa Fluor 647 donkey anti-rabbit (Invitrogen, Burlington, ON, Canada).

### 4.3. Proximity Dependent Biotinylation

BioID and mass spectrometry were conducted according to the protocol from Coyaud et al. (Mol. Cell. Proteomics 2015) [83]. Cells were grown in five 15 cm cell culture dishes until 70% confluence. Cells were incubated for 24 h in complete media supplemented with 1 µg/mL tetracycline (BioShop, Burlington, ON, Canada) and 50 µM biotin (BioShop, Burlington, ON, Canada) 8 h post initial induction. Cells were lysed, sonicated twice for 10 s at 35% amplitude (Sonic Dismembrator 500; Fisher Scientific, Waltham, MA, USA) and centrifuged at 16,000 rpm (35,000× *g*) for 30 min at 4 °C. Supernatants were passed through a Micro Bio-Spin Chromatography column (Bio-Rad 732-6204, Hercules, CA, USA) and incubated with 30µL of high-performance streptavidin-packed beads (GE Healthcare, Chicago, IL, USA) for 3 h at 4 °C on an end-over-end rotator. Beads were collected (2000 rpm, 2 min) and washed six times with 50 mm ammonium bicarbonate (pH8.3). Beads were then treated with L-1-Tosylamide-2-phenylethyl chloromethyl ketone (TPCK)-treated trypsin (Promega, Madison, WI, USA) for 16 h at 37 °C on an end-over-end rotator. Another 1 µL of TPCK-trypsin was added and incubated in a water bath at 37 °C for 2 h. Supernatants were lyophilized and stored at 4 °C for downstream mass spectrometry analysis.

### 4.4. Experimental Design and Statistical Rationale

Four BioID runs were conducted on FlagBirA*-CFTR WT and mutant lines. These four runs consisted of two technical replicates (*n* = 2) from two biological replicates (*n* = 2; total *n* = 4). Control runs of a BioID analysis conducted on the corresponding fractions on cells expressing the FlagBirA*-tag alone were used for comparative purposes. Replicates were completed for FlagBirA*-CFTR in vehicle control (0.1% DMSO) conditions. Replicates were completed for FlagBirA*-∆F508- CFTR in both vehicle control (0.1% DMSO) and drug exposed (3 µM VX-809 + 1 µM VX-770) conditions, in the presence or absence of the proteasome inhibitor, MG132. Data were analyzed using the trans-proteomic pipeline via the ProHits 5.0.2 software suite. Proteins identified with an iProphet cut-off of 0.9 were analyzed using SAINT Express v. 3.6.1 [83] to identify high confidence interactors (MSV000088626). BioID datasets were highly reproducible. All replicates for each condition were tested for correlation and ensured to have an average R^2^ value > 0.9 before proceeding with the analysis (Supplementary Figures S6–S10). Proteins identified that scored above a Bayesian False Discovery Rate (BFDR) of 1% were considered high confidence interactors. Normalization of prey spectral counts was implemented using bait spectral counts for each condition when comparing datasets (Supplementary Figure S3b). Proximity interactors considered significantly gained or lost upon exposure to combination therapy had to achieve a log_2_-fold change (log_2_FC) ± 1.0. The logarithmic ratio of protein intensities between two samples and the negative logarithmic *p*-values of the Student’s *t*-test obtained from biological replicates between samples were calculated for volcano plot analysis. Volcano plot compared biotinylated proteins identified in ∆F508 + MG132 vehicle to Orkambi exposed conditions illustrating preys enriched in GO categories for ER, chaperone, protein folding, and cellular response to stress. Preys marked in red have lower spectral counts in the ∆F508 + MG132 + Orkambi condition (log_2_FC < −0.4). Preys marked in green have higher spectral counts in the ∆F508 + MG132 + Orkambi condition (log_2_FC < 0.4).

### 4.5. CFTR Channel Function in CFTR expressing HEK293 Cells

CFTR FLAG-BirA* fusions were expressed in HEK293 Flp-In T-REx cells and were seeded in 96-well plates (Costar, Corning). After 24 h tetracycline induction, cells were then loaded with blue FLIPR membrane potential dye dissolved in chloride-free buffer (136 mM sodium gluconate, 3 mM potassium gluconate, 10 mM glucose, 20 mM HEPES, pH 7.35, 300 mOsm, at a concentration of 0.5 mg/mL) for 30 min at 37 °C. CFTR function was determined using BioTek Synergy HTX Multi-Mode Reader at 37 °C. After establishing a baseline fluorescence read (excitation 530 nm/emission 560 nm) for 3 min, CFTR was stimulated using Forskolin (Fsk) (10 µM, MedChemExpress, Princeton, NJ, USA). CFTR-mediated depolarization of the plasma membrane was detected as an increase in fluorescence following which the CFTR inhibitor, CFTRinh-172 (10 µM, MedChemExpress, Princeton, NJ, USA) was added to inactivate CFTR. The changes in fluorescence to CFTR agonist were normalized relative to the average baseline fluorescence (∆F/F0) [84].

### 4.6. SiRNA KD of Candidate Interactors

CFTR siRNA was purchased from Ambion (Austin, TX, USA) and designed to target CFTR. Previously validated VAPB siRNA [85] was kindly provided by Dr. Peter Kim’s laboratory (Toronto, ON, Canada). siRNAs were transfected at 20 nM using RNAiMAX (Invitrogen/Thermofisher, Burlington, ON, Canada) and following the manufacturers’ instructions. After 48 h, CFTR expression was induced by supplementing the media with 1 µg/mL tetracycline, and cells were used in downstream assays after an additional 24 h.

### 4.7. Surface Expression Assay and Image Analysis

After siRNA transfection, CFBE mCherry-Flag-WT-CFTR cells were seeded on custom patterned coverslips [86]. After 72 h, extracellular Flag-tags were immunostained in non-permeabilized cells. After culture medium removal, cells were washed once in ice cold PBS and incubated 45 min on ice with anti-FLAGm2 antibody. Then, cells were washed 3 times with ice cold PBS, incubated 10 min with 4% PFA on ice and transferred to room temperature for the remaining staining procedure. Cells were washed with PBS and incubated 30 min with Alexa Fluor 488 donkey anti-mouse before mounting onto glass slides. Fluorescence images were acquired on an automated DeltaVision Microscope with a 60× 1.4NA objective and 2 × 2 binning (GE Healthcare). For every well, 25 fields of Z-stacks encompassing 8 µm were deconvolved, projected and exported as 16-bit TIFF images prior to analysis. On average, each field sampled 25–40 cells. Using the MATLAB image analysis toolbox, we estimated dark noise and background using demarcated regions in several images from each dataset. The background was calculated using the most populated pixel bin from histograms of these regions (for each channel), and subtracted from the corresponding channels. For every field, each channel was thresholded using a stringent cutoff (7× and 20× over background for the ‘surface’ and ‘total’ channels, respectively) to select only pixels corresponding to cellular contents, and all other pixel values were discarded from subsequent calculations. The mean thresholded pixel intensity for each channel was then calculated. The ratio of surface:total was calculated by dividing the two background-subtracted and thresholded ‘surface’ and ‘total’ channels for every field, thereby generating 25 ratios for each set. All imaging experiments were performed three times and displayed similar trends. The mean and standard error of three experiments is plotted (Figure 5d). MATLAB scripts are available upon request.

### 4.8. BioInformatics and Data Visualization

Gene Ontology (GO) enrichments were performed using PANTHER Classification system v.16.0. The CFTR dataset included all proteins defined in the SAINT output file. Known interactions for CFTR were downloaded from BioGRID77 (version 4.4.203). The networks were generated using Cytoscape79 version 3.8.0. Dot plots were generated using ProHits-viz78 [87] Quantitation is encoded using the color gradient representing control-subtracted spectral counts (capped at 20), with relative spectral counts across baits represented by node size. Border colour is encoded by BFDR value (black ≤ 0.01; blue ≤ 0.05; light blue > 0.05). Prey profiles were compared to the curated BioID dataset of 192 cellular markers [37] and the Jaccard Distances between datasets were exported for analysis. Interactors for which no meaningful function or localization was found via protein databases [88] were removed.

## Data Availability

The data presented in this study are available in the Supplementary Materials, raw MS data are available at MSV000088626.

## Supplementary Materials

The supporting information can be downloaded at: https://www.mdpi.com/article/10.3390/ijms23052442/s1.
