# Supplementary material for: Proximity Profiling of the CFTR Interaction Landscape in Response to Orkambi
Source: Int J Mol Sci. 2022 Feb 23;23(5):2442. doi: 10.3390/ijms23052442 (PMC8910062; doi:10.3390/ijms23052442)
Supplement: Supplementary file 1 [file ijms-23-02442-s001.zip › Supplementary Figures.pdf]

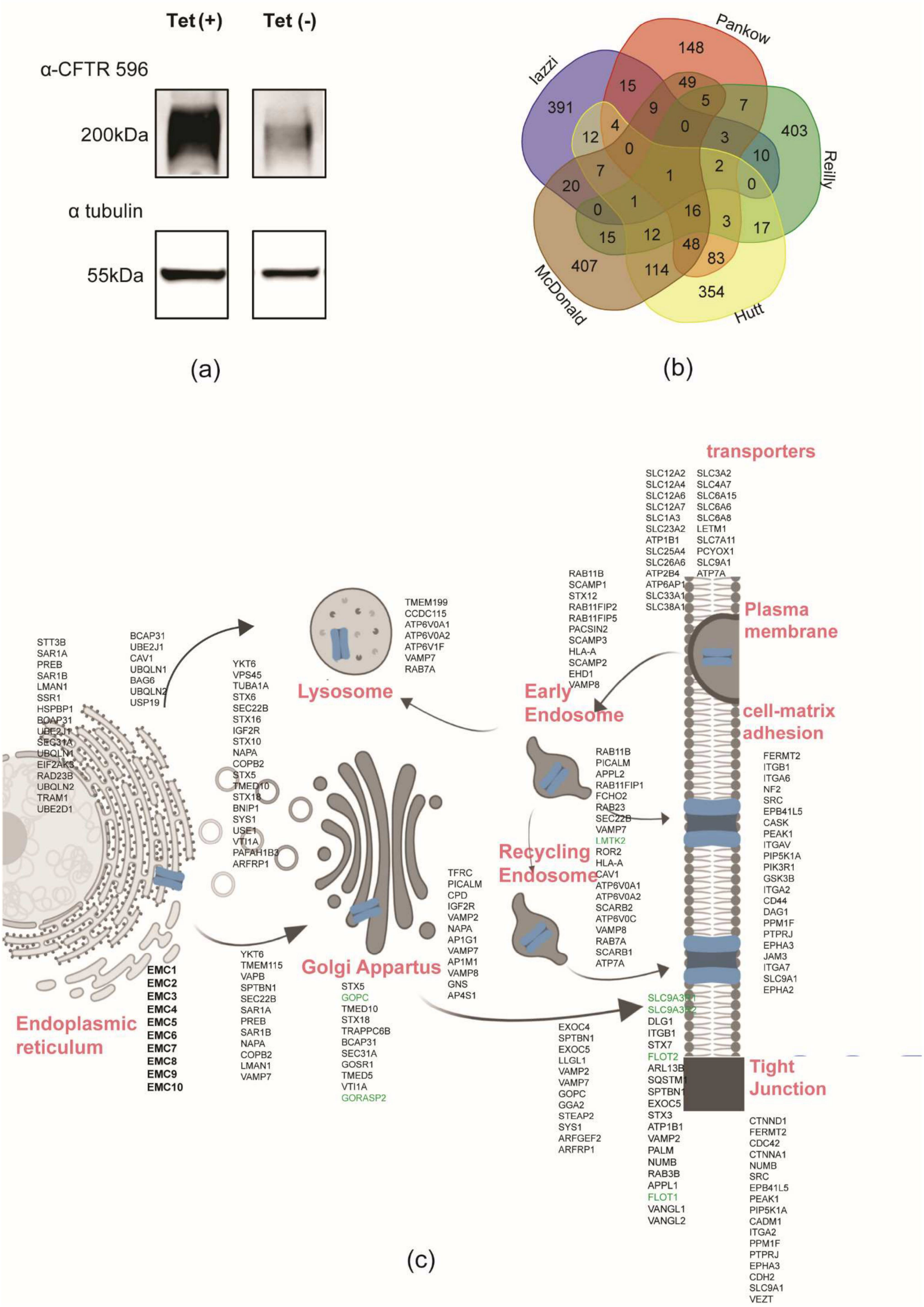

Supplementary Figure S1. (a) HEK293 T-REx cells stably expressing FLAG-BirA*-CFTR were analyzed after 24-**h** induction with tetracycline (1μM). WB performed using whole cells confirms successful expression of WT CFTR in induced conditions. (b) Overlap of the BioID WT-CFTR interactome with previously generated CFTR interactomes. (c) CFTR processing map as revealed by proximity profiling. The map represents our interpretation of the location and biotin labelling of the CFTR-BirA* protein (blue). Green indicates known

interactors of CFTR. Preys identified show that FLAG-BirA*-CFTR follows its biosynthetic route from the ER to the PM and enters in recycling and degradative routes. Interactors for which no meaningful function or localization was found via protein databases [88] were removed.

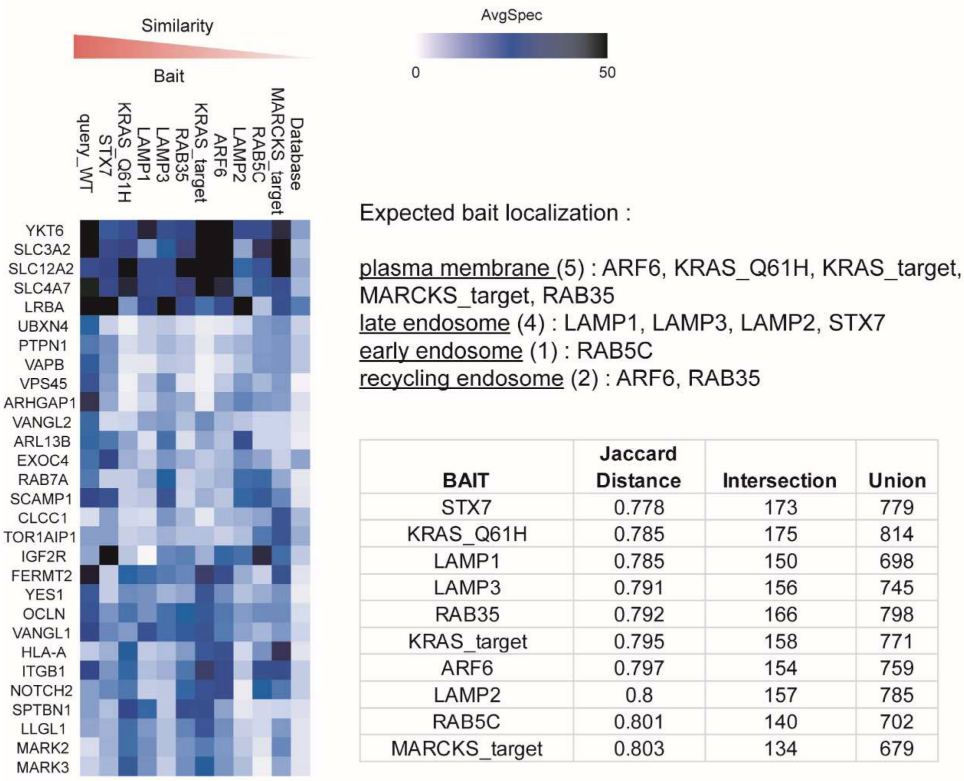

Expected bait localization :

plasma membrane (5) : ARF6, KRAS_Q61H, KRAS_target, MARCKS_target, RAB35
late endosome (4) : LAMP1, LAMP3, LAMP2, STX7
early endosome (1) : RAB5C
recycling endosome (2) : ARF6, RAB35

BAIT	Jaccard Distance	Intersection	Union
STX7	0.778	173	779
KRAS_Q61H	0.785	175	814
LAMP1	0.785	150	698
LAMP3	0.791	156	745
RAB35	0.792	166	798
KRAS_target	0.795	158	771
ARF6	0.797	154	759
LAMP2	0.8	157	785
RAB5C	0.801	140	702
MARCKS_target	0.803	134	679

(a)

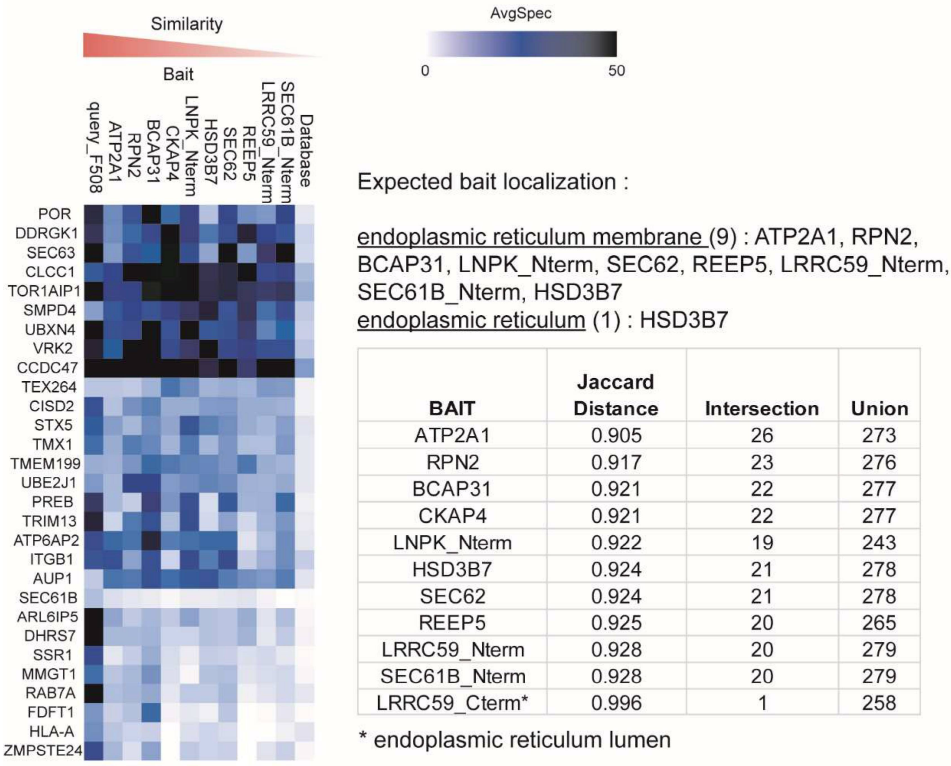

Expected bait localization :

endoplasmic reticulum membrane (9) : ATP2A1, RPN2, BCAP31, LNPK_Nterm, SEC62, REEP5, LRRC59_Nterm, SEC61B_Nterm, HSD3B7
endoplasmic reticulum (1) : HSD3B7

BAIT	Jaccard Distance	Intersection	Union
ATP2A1	0.905	26	273
RPN2	0.917	23	276
BCAP31	0.921	22	277
CKAP4	0.921	22	277
LNPK_Nterm	0.922	19	243
HSD3B7	0.924	21	278
SEC62	0.924	21	278
REEP5	0.925	20	265
LRRC59_Nterm	0.928	20	279
SEC61B_Nterm	0.928	20	279
LRRC59_Cterm*	0.996	1	258

* endoplasmic reticulum lumen

(b)

Supplementary Figure S2. Comparison of WT-CFTR (a) and ΔF508-CFTR (b) BioID interactomes with 192 BioID baits representing all major cellular compartments [37]. The spectral abundance heat map (see legend on the top of the heat map) in the left panels represents a portion of the overlapping preys that are present in baits with the most similar profiles to the query. The panel on the right is a prediction of the query bait's localization based on its similarity to the 192 BioID bait profiles that represent known cellular compartment markers. The table outlines the pairwise Jaccard Distance between the query bait and the comparison cellular marker bait profiles. The WT-CFTR bait profile is most aligned with plasma membrane and membrane trafficking markers (a), while the ΔF508-CFTR profile overlaps best with membrane proteins in the endoplasmic reticulum tagged at their cytoplasmic, but not lumenal domains (b).

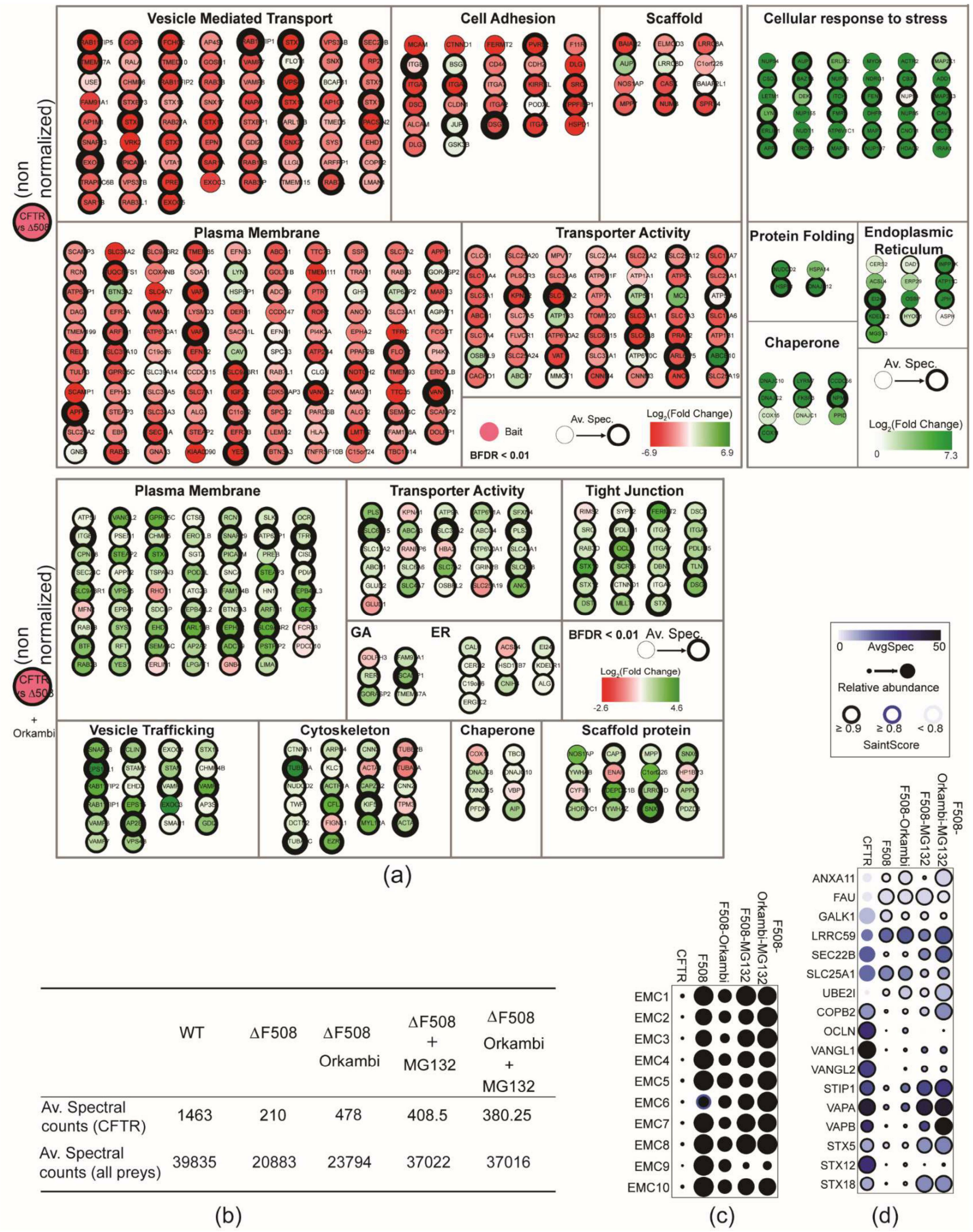

Supplementary Figure S3. (a) CFTR interactome filtered 'preys' with a BFDR ≤ 0.01. Preys categorized using Gene Ontology (GO) enrichment for key cellular components or functions. The thickness of the border represents

an increasing aver-age peptide count. The node colour reflects the $\log_2$ fold change from WT to the non-normalized ΔF508 mutant condition (top panel) or from WT to the non-normalized ΔF508 + Orkambi condition (bottom panel). The darker red nodes represent greater negative fold change. The darker green nodes represent greater positive fold change. (b) Spectral counts of self-labelled FLAG-BirA*-CFTR in all bait conditions; FLAG-BirA*-CFTR, FLAG-BirA*-ΔF508-CFTR in vehicle and Orkambi exposed conditions, with and without MG132. CFTR peptide spectral counts increased ~2x upon MG132 addition. Total average spectral counts summed from all preys pulled down in each bait condition. (c) The Prohits-viz web tool [87] was used to generate this dot plot, displaying prey abundance across baits and prey confidence. All 10 EMC subunits were identified in each of the 5 WT and ΔF508-CFTR datasets. Prey spectral counts in ΔF508-CFTR in vehicle and Orkambi conditions were normalized to bait CFTR spectral counts. (d) Dot plot of a subset of the enriched mutant preys in GO categories for ER, chaperone, protein folding, and cellular response to stress. The counts are shown un-normalized in order to compare to the normalized version in Figure 4b. Note that some prey counts fall below the threshold of SAINT score significance but are included for reference.

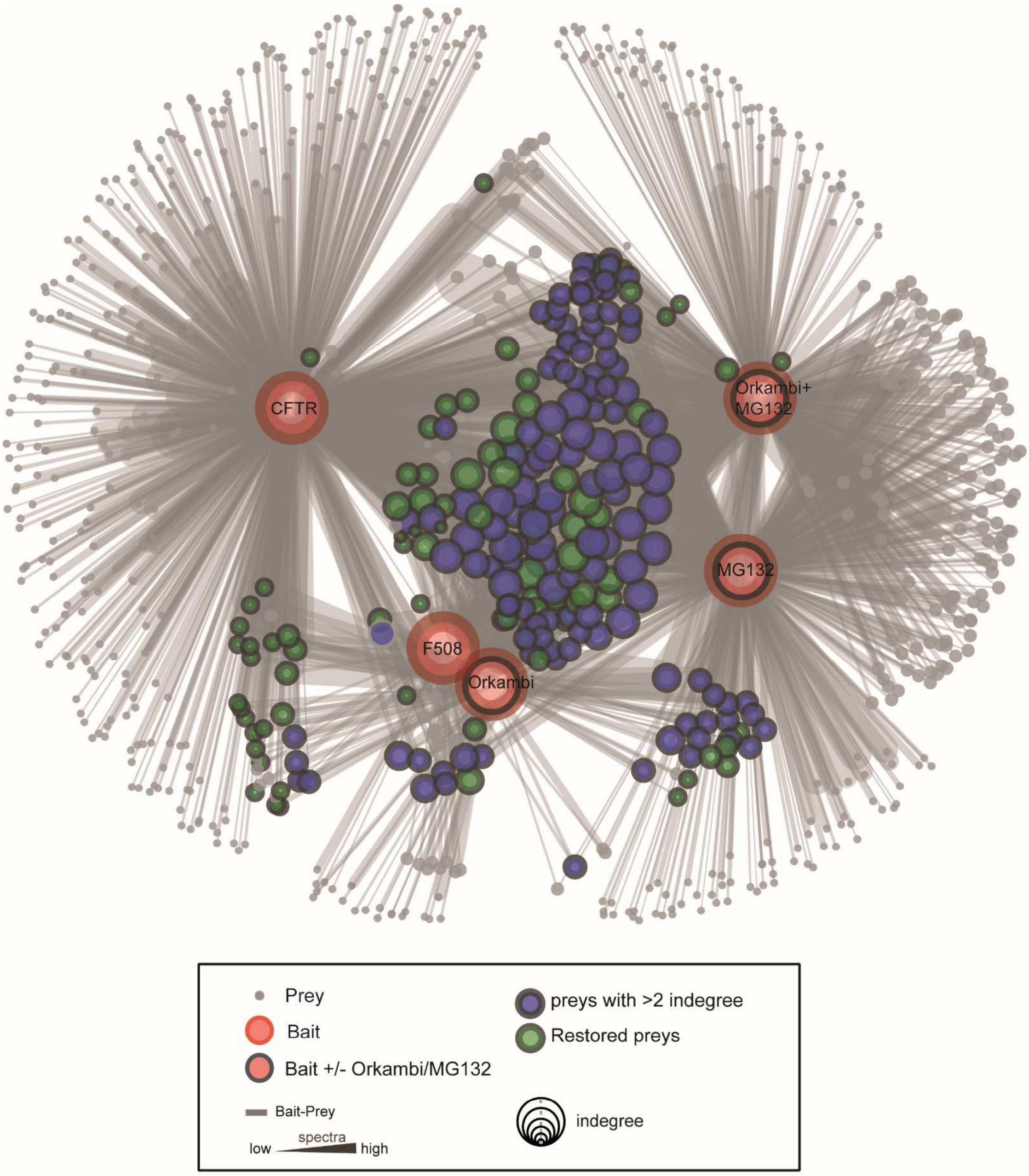

Supplementary Figure S4. The CFTR proximity interactome. Unsupervised, edge-weighted spring embedded view of FLAG-BirA*-CFTR WT and mutant profiles Orkambi/MG132 treatment. The size of each 'prey' is drawn differently depending on how many 'baits' it is connected to (indegree). Preys common to all baits cluster into the middle. The topological representation reveals that a subset of F508 mutant backgrounds (green) 'pulled' towards the WT bait after pharmacotherapy.

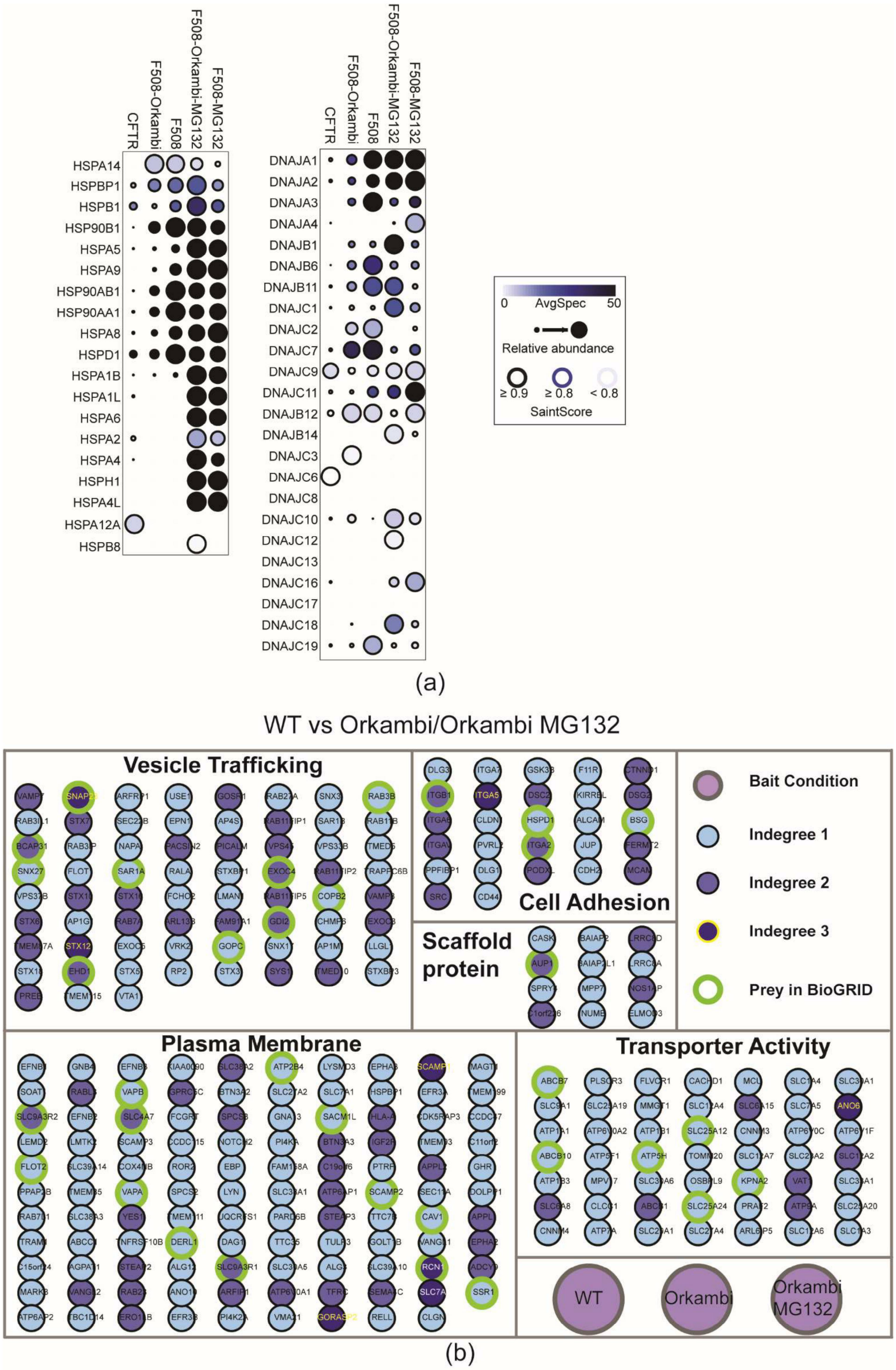

Supplementary Figure S5. (a) Dot plots of normalized prey abundance of Hsp chaperone components- Hsp 70/90 (left panel) and DNAJ (right panel)- across all bait conditions. (b) Overlap of WT CFTR (WT) and Orkambi restored interactors. WT high confidence interactors (BFDR ≤ 0.01) and significantly restored interactors (log_2FC

≥ 1) from ΔF508 vehicle to exposure of Orkambi or Orkambi + MG132. Indegree 1 represents a prey detected by only the WT bait, while Indegrees 2 and 3 are preys detected with either one or both of Orkambi and Orkambi+MG132 treated baits respectively. Known CFTR-interactors identified using BioGRID databases are bordered in green.

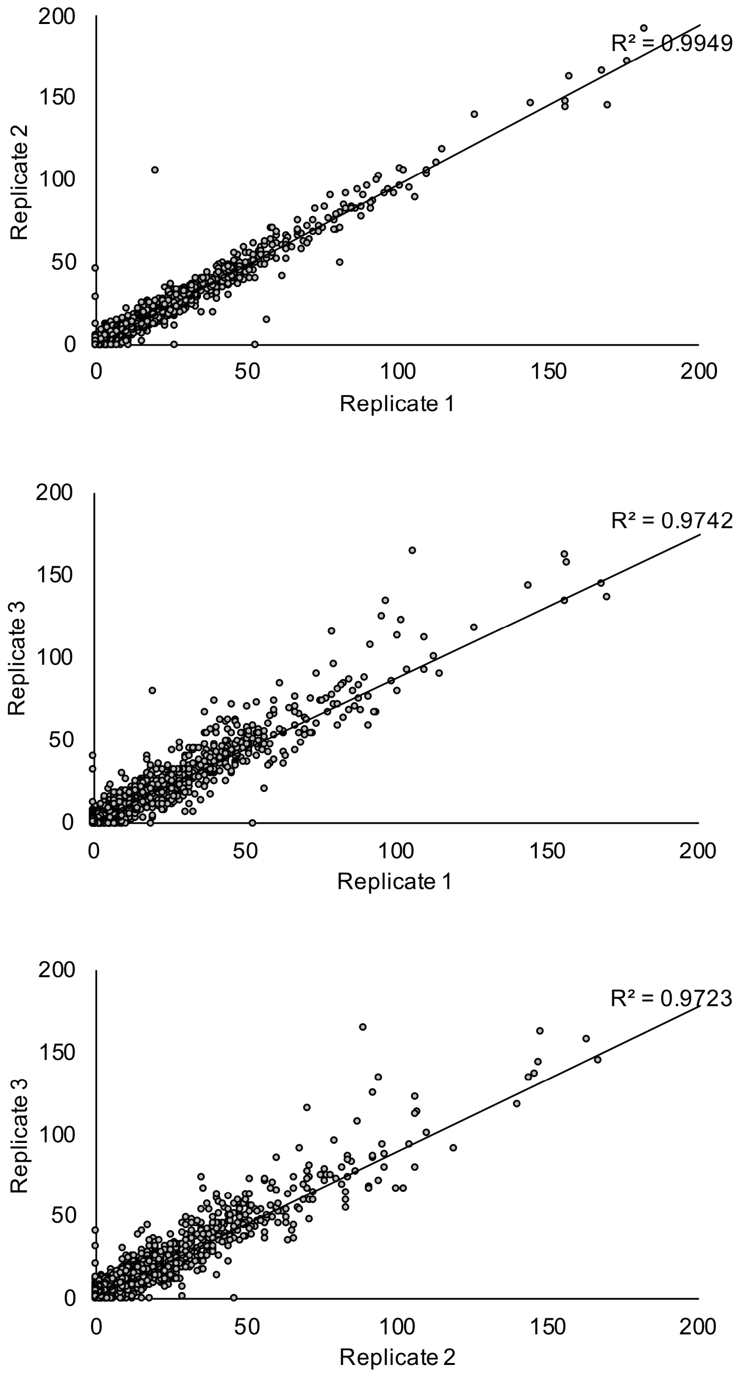

(a)

	Rep1	Rep2	Rep3
**Rep1**	**X**	0.9949	0.9742
**Rep2**	0.9949	**X**	0.9723
**Rep3**	0.9742	0.9723	**X**

(b)

Supplementary Figure S6. FLAG-BirA*-CFTR BioID. (a) Scatter plots comparing spectral counts for every prey different replicate runs of the HEK293 CFTR BioID dataset. (b) Summary of R^2 values generated from comparisons between each replicate run.

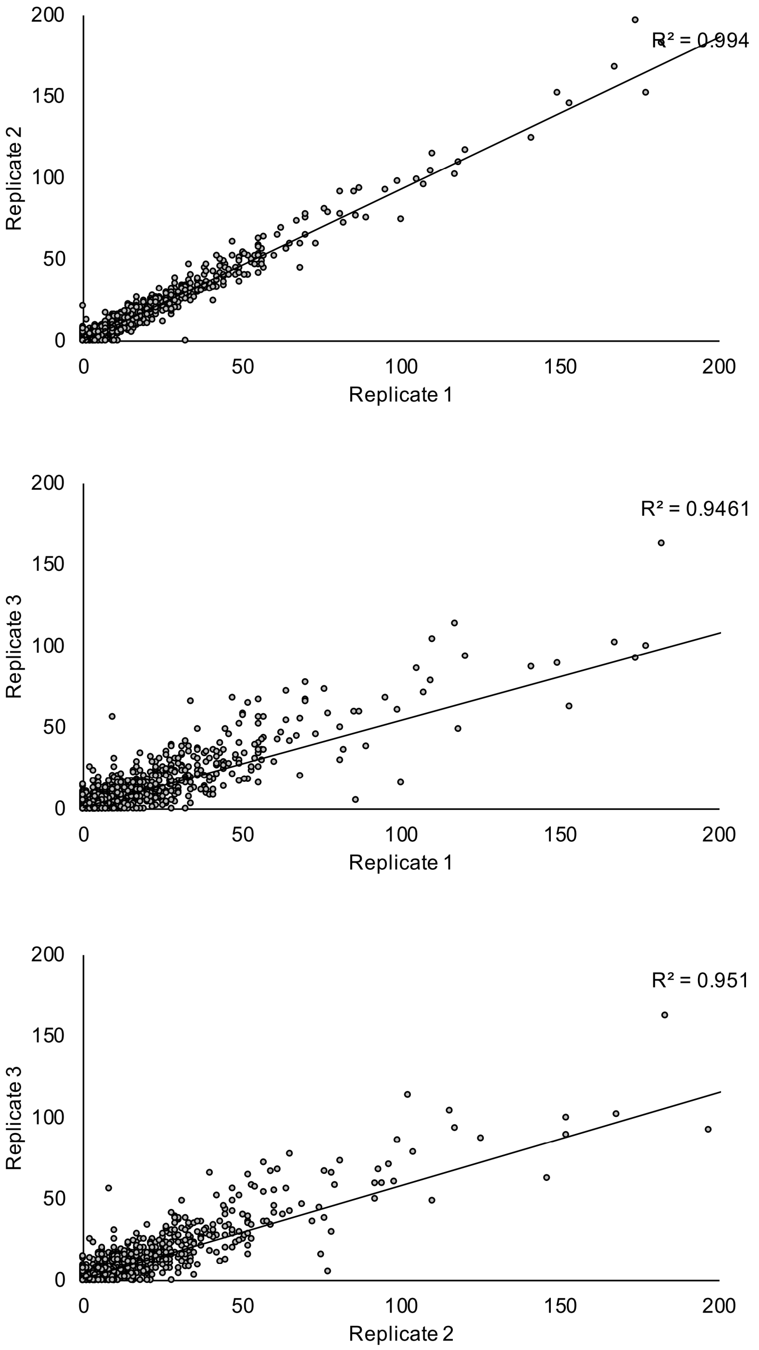

(a)

	**Rep1**	**Rep2**	**Rep3**
**Rep1**	**X**	0.9940	0.9461
**Rep2**	0.9940	**X**	0.9510
**Rep3**	0.9461	0.9510	**X**

(b)

Supplementary Figure S7. FLAG-BirA*-ΔF508-CFTR BioID. (a) Scatter plots comparing **Scheme 293.** FLAG-BirA*-ΔF508-CFTR + MG132 BioID dataset. (b) Summary of R^2 values generated from comparisons between each replicate run. .

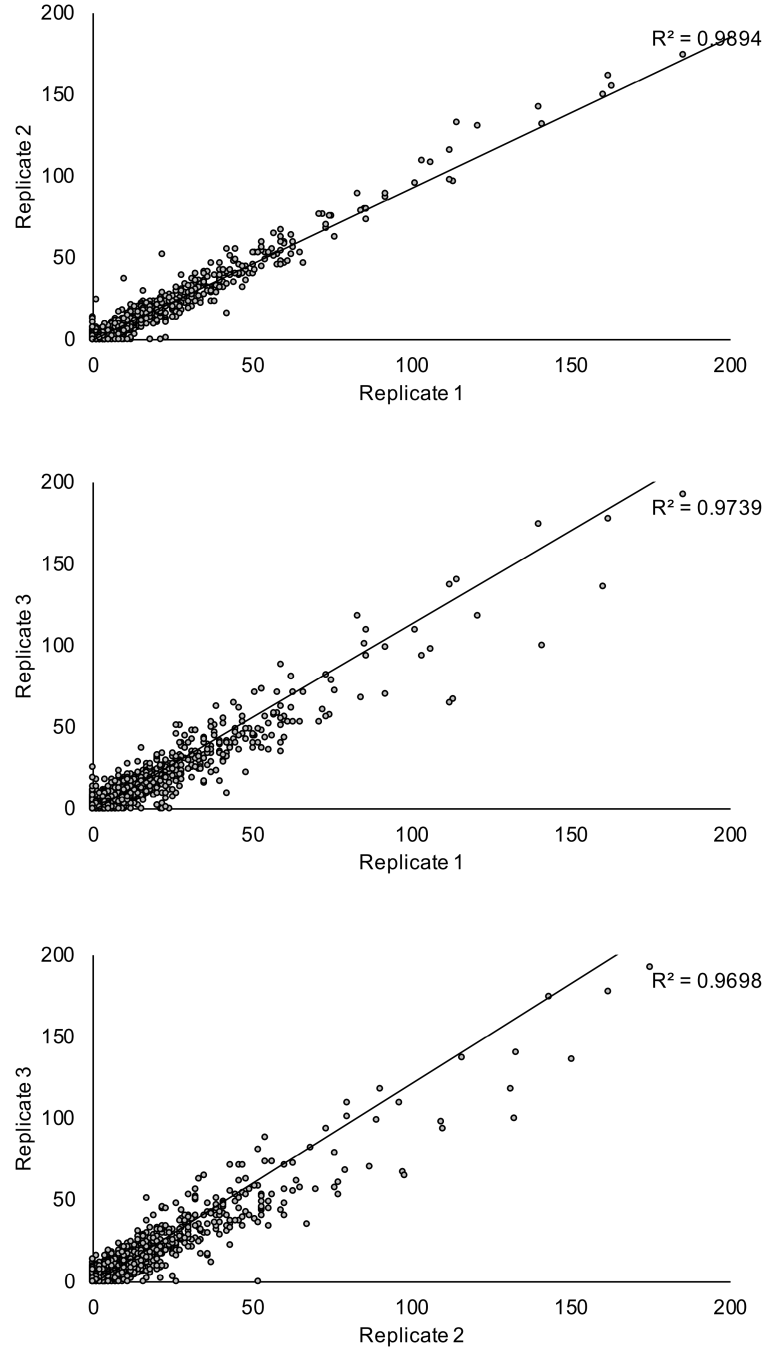

(a)

	Rep1	Rep2	Rep3
**Rep1**	**X**	0.9894	0.9739
**Rep2**	0.9894	**X**	0.9698
**Rep3**	0.9739	0.9698	**X**

(b)

Supplementary Figure S8. FLAG-BirA*-ΔF508-CFTR Orkambi BioID. (a) Scatter plots comparing spectral counts for every prey different replicate runs of the HEK293 FLAG-BirA*-ΔF508-CFTR +

MG132 BioID dataset. (b) Summary of R^2 values generated from comparisons between each replicate run.

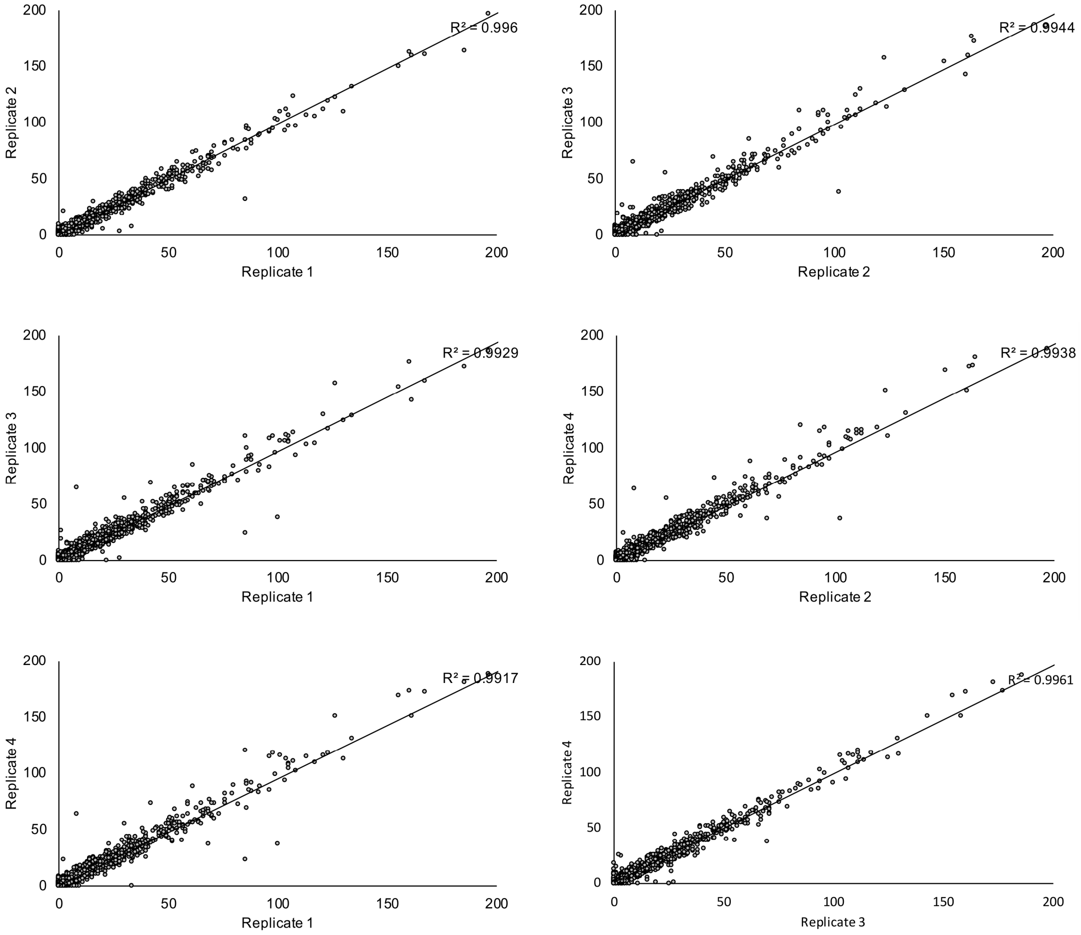

(a)

	Rep1	Rep2	Rep3	Rep4
**Rep1**	**X**	0.9960	0.9929	0.9917
**Rep2**	0.9960	**X**	0.9944	0.9938
**Rep3**	0.9929	0.9944	**X**	0.9961
**Rep4**	0.9917	0.9938	0.9961	**X**

(b)

Supplementary Figure S9. FLAG-BirA*-ΔF508-CFTR + MG132 BioID . (a) Scatter plots comparing spectral counts for every prey different replicate runs of the HEK293 FLAG-BirA*-ΔF508-CFTR + MG132 BioID dataset. (b) Summary of R^2 values generated from comparisons between each replicate run.

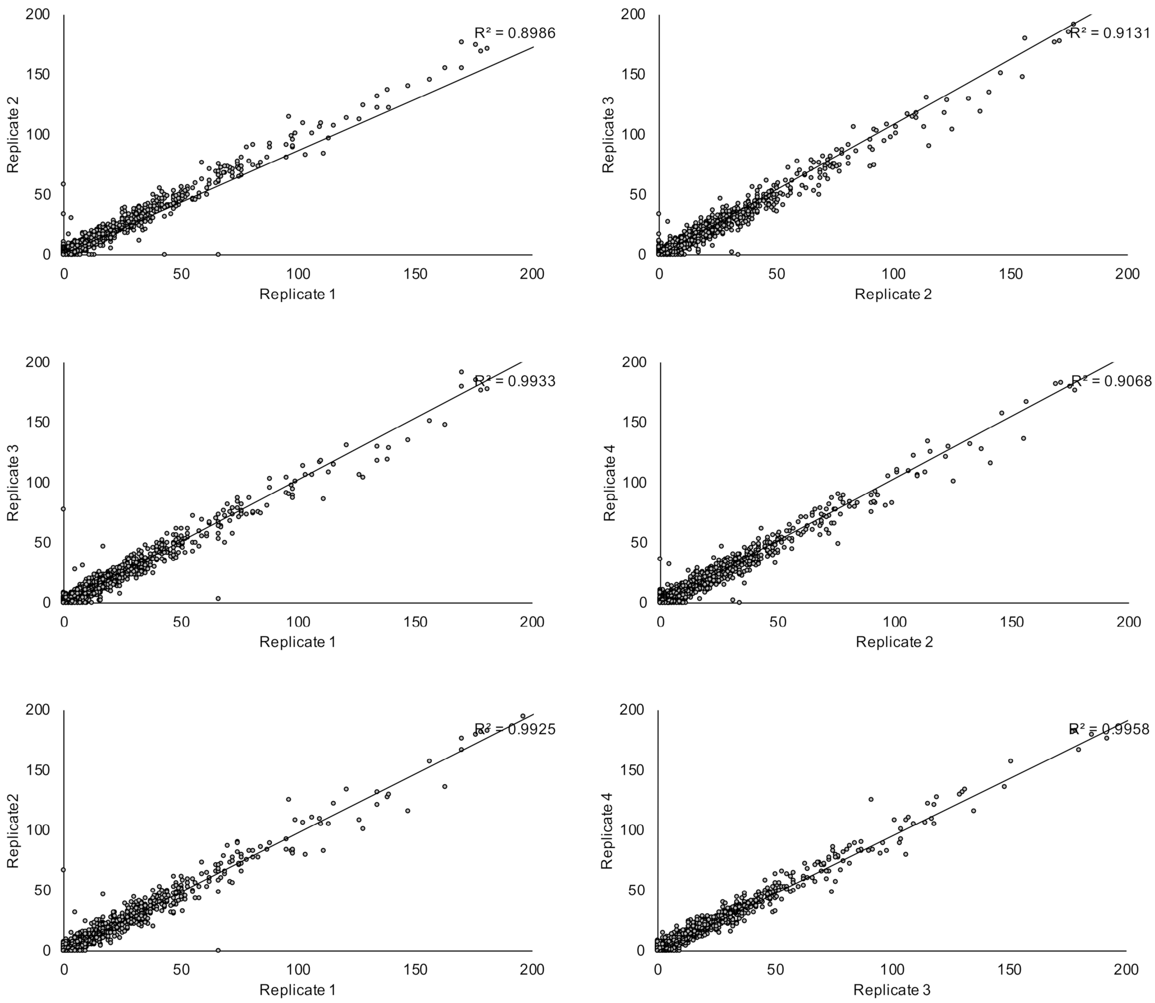

(a)

	Rep1	Rep2	Rep3	Rep4
**Rep1**	**X**	0.8986	0.9933	0.9925
**Rep2**	0.8986	**X**	0.9131	0.9068
**Rep3**	0.9933	0.9131	**X**	0.9958
**Rep4**	0.9925	0.9068	0.9958	**X**

(b)

Supplementary Figure S10. FLAG-BirA*-ΔF508-CFTR Orkambi + MG132 BioID. (a) Scatter plots comparing spectral counts for every prey different replicate runs of the HEK293 FLAG-BirA*-ΔF508-CFTR + MG132 BioID dataset. (b) Summary of R^2 values generated **Figure .** rom comparisons between each replicate run.
